# Differential heparan sulfate dependency of the *Drosophila* glypicans

**DOI:** 10.1016/j.jbc.2023.105544

**Published:** 2023-12-10

**Authors:** Eriko Nakato, Keisuke Kamimura, Collin Knudsen, Suzuka Masutani, Masahiko Takemura, Yoshiki Hayashi, Takuya Akiyama, Hiroshi Nakato

**Affiliations:** 1 Department of Genetics, Cell Biology, and Development, University of Minnesota, Minneapolis, Minnesota, USA; 2 Developmental Neuroscience Project, Department of Brain and Neurosciences, Tokyo Metropolitan Institute of Medical Science, Tokyo, Japan; 3 Life Science Center for Survival Dynamics, Tsukuba Advanced Research Alliance (TARA), University of Tsukuba, Tsukuba, Japan; 4 Department of Biology, Indiana State University, Terre Haute, Indiana, USA

**Keywords:** heparan sulfate, proteoglycan, glypican, morphogen, *Drosophila*

## Abstract

Heparan sulfate proteoglycans (HSPGs) are composed of a core protein and glycosaminoglycan (GAG) chains and serve as coreceptors for many growth factors and morphogens. To understand the molecular mechanisms by which HSPGs regulate morphogen gradient formation and signaling, it is important to determine the relative contributions of the carbohydrate and protein moieties to the proteoglycan function. To address this question, we generated ΔGAG alleles for *dally* and *dally-like protein* (*dlp*), two *Drosophila* HSPGs of the glypican family, in which all GAG-attachment serine residues are substituted to alanine residues using CRISPR/Cas9 mutagenesis. In these alleles, the glypican core proteins are expressed from the endogenous loci with no GAG modification. Analyses of the *dally*^*ΔGAG*^ allele defined Dally functions that do not require heparan sulfate (HS) chains and that need both core protein and HS chains. We found a new, *dally*^*ΔGAG*^-specific phenotype, the formation of a posterior ectopic vein, which we have never seen in the null mutants. Unlike *dally*^*ΔGAG*^, *dlp*^*ΔGAG*^ mutants do not show most of the *dlp* null mutant phenotypes, suggesting that HS chains are dispensable for these *dlp* functions. As an exception, HS is essentially required for Dlp's activity at the neuromuscular junction. Thus, *Drosophila* glypicans show strikingly different levels of HS dependency. The *ΔGAG* mutant alleles of the glypicans serve as new molecular genetic toolsets highly useful to address important biological questions, such as molecular mechanisms of morphogen gradient formation.

Precise control of cell fate decisions is a crucial step of tissue morphogenesis. The secreted signaling molecules called morphogens form concentration gradients in a developing tissue and specify the cell fate in a concentration-dependent manner. In the *Drosophila* developing wing, three major morphogens play key roles. Hedgehog (Hh) is expressed in the posterior compartment and induces expression of target genes in the anterior compartment. One of the Hh target genes is another morphogen, Decapentaplegic (Dpp; a *Drosophila* bone morphogenetic protein [BMP]). In addition, Wingless (Wg) is expressed in a stripe of cells at the dorsoventral compartment boundary and regulates patterning along the D-V axis.

Heparan sulfate proteoglycans (HSPGs), a class of carbohydrate-modified proteins, play essential roles in morphogen signaling and gradient formation (1, 2, 3, 4, 5). For example, Dally, a *Drosophila* HSPG of the glypican family, acts as a coreceptor for Dpp (6, 7, 8, 9). Another glypican, Dally-like protein (Dlp), is not a major regulator of Dpp (7, 10). Instead, Dlp is an essential Hh coreceptor (11, 12, 13, 14, 15). Both glypicans regulate Wg signaling, but they play distinct functions in the pathway (16, 17, 18). Thus, the two glypicans, Dally and Dlp, are thought to be functionally distinct, sharing only limited functional redundancy. The molecular mechanisms of the coreceptor activity and functional specificity between different HSPGs remain important questions.

Morphogen signaling pathways form multiple feedback loops to buffer against genetic and environmental perturbations, and HSPG coreceptors are major components of such feedback systems (19, 20). For example, expression of Dally, which enhances Dpp signaling as a coreceptor, is repressed by Dpp signaling (6). Thus, Dally forms a negative feedback loop of this pathway. Similarly, *dally* expression is also controlled by Wg and Hh signaling, two additional pathways Dally regulates (21). The heparan sulfate (HS) modification machinery also plays a role in the morphogen feedback systems. Sulf1 is a secreted HS endosulfatase and negatively regulates Wg signaling by removing ligand-binding sites on HS (22). Expression of *Sulf1* is induced by the Wg pathway itself. A similar phenomenon has also been reported for the Hh and Vein-epidermal growth factor receptor pathways (23, 24). In addition, it is known that a loss of one type of HS sulfation is compensated for by an increase in sulfation at different positions (25, 26, 27). Thus, feedback loops of HSPGs and HS sulfation control provide compensatory effects to minimize consequences of a genetic change in morphogen signaling, contributing to the robustness of the system (9, 20).

HSPGs are composed of HS chains that are attached to specific serine residues of the core proteins. It is clear that HS chains are critical for HSPG coreceptor activity because blocking HS biosynthesis inhibits morphogen signaling. Virtually all signaling events mediated by fibroblast growth factors, Dpp, Wg, and Unpaired (Upd; a ligand of the Jak/Stat pathway) are disrupted in HS-deficient animals, such as *tout-velu* (*ttv*; a *Drosophila* homologue of Ext proteins, which functions as a HS copolymerase) and *sulfateless* (*sfl*; the only *Drosophila* homologue of HS N-deacetylate/N-sulfotransferase, a key enzyme for HS modifications) (28, 29, 30). On the other hand, core protein structures of syndecans and glypicans are highly conserved during evolution (31), suggesting the importance of the core proteins in signaling activity. Biochemical and structural studies have demonstrated direct interactions between HSPG core proteins and morphogen ligands ((15, 16, 32, 33, 34)). Thus, the core protein plays a significant role and is not just as a carrier of HS chains. Therefore, it is important to understand the relative contribution of the sugar and protein moieties to proteoglycan functions.

In *Drosophila* genetics, the Gal4/UAS system using a “*UAS-PG*^*ΔGAG*^” construct has been used as the major approach to address this question (12, 35, 36, 37). The *UAS-PG*^*ΔGAG*^ constructs were made by substituting the glycosaminoglycan (GAG)-attachment serine residues with alanine residues so that HS cannot be added to the core protein. By overexpressing wildtype *PG* and *PG*^*ΔGAG*^ with the Gal4/UAS system, the *in vivo* activities of the two constructs and their ability to rescue proteoglycan mutant phenotypes can be compared. Although this method has been helpful to determine the roles of HS chains and the core proteins, it has some limitations. Since there is no perfect Gal4 driver, the results tend to include unwanted effects of overexpression and ectopic expression. Given that morphogen signaling is a highly quantitative system, the Gal4-driven overexpression does not provide an appropriate platform to precisely assess the coreceptor activities. Namely, a previous work has shown that *PG*^*ΔGAG*^ overexpression interferes with HS modification of other proteins, which makes it difficult to interpret data (35). Therefore, an ideal strategy to address this question is to make an endogenous Δ*GAG* allele by genome editing.

In this study, we generated and performed phenotypic analyses of ΔGAG alleles for *dally* and *dlp*. We found that these glypicans show strikingly different levels of HS dependency. Our analyses of double mutants, *dally*^*gem*^ *dlp*^*ΔGAG*^ and *dally*^*ΔGAG*^ *dlp*^*ΔGAG*^, raised a possibility that the loss of HS chains of glypicans activates compensatory feedback mechanisms.

## Results

### Δ*GAG* alleles of *dally* and *dlp* genes

**Figure 1:**
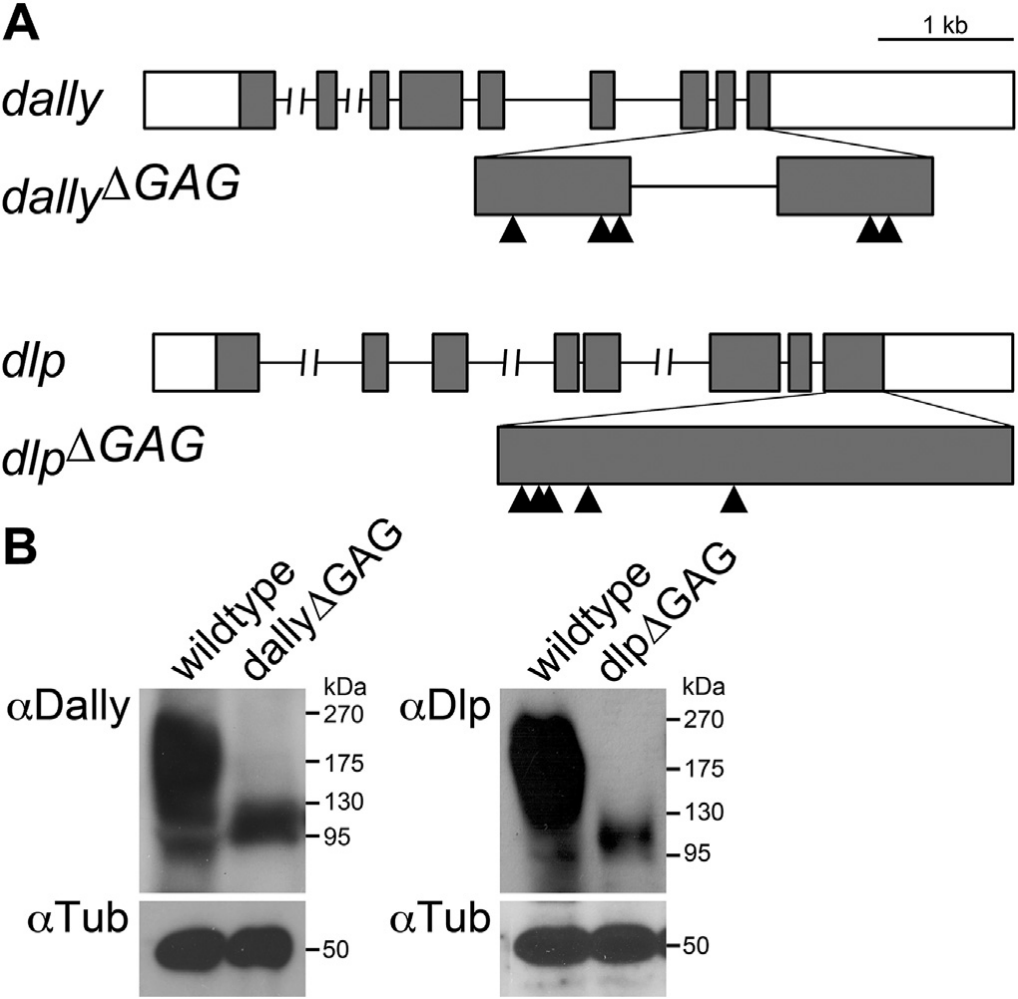
**ΔGAG alleles of *dally* and *dlp* genes.***A*, a schematic of the *dally* and *dlp* loci. *Arrowheads* indicate the position of serine residues that are substituted to alanine residues. Amino acid sequences of Dally^ΔGAG^ and Dlp^ΔGAG^ are shown in Fig. S1. *B*, immunoblot analysis of ΔGAG alleles. Protein extracts from wildtype, *dally*^*ΔGAG*^, and *dlp*^*ΔGAG*^ adult flies were analyzed using anti-Dally (*left*) and anti-Dlp (*right*) antibodies (*top panels*). Anti-α-Tubulin antibody was used as an internal control (*bottom panels*).

Using CRISPR/Cas9 mutagenesis, we generated *ΔGAG* alleles for the two *Drosophila* glypicans, Dally and Dlp (Fig. 1*A*). In both loci, all GAG-attachment serine residues were substituted with alanine residues. The positions of the mutated residues are shown by arrowheads in Figure 1, and the specific serine residues altered in the entire amino acid sequences are shown in Fig. S1. In these alleles, the glypican core proteins are expressed from their endogenous loci with no GAG modification. In the Western blot analyses of wildtype extract, anti-Dally and anti-Dlp antibodies detect high-molecular-weight proteins as smear bands. These smear bands are characteristic of proteoglycans reflecting varying lengths of individual GAG chains (Fig. 1*B*). These smear bands disappear in the *ΔGAG* mutant extracts, confirming the lack of GAG modification.

### *dally*^*ΔGAG*^ shows normal patterning of notal mechanosensory bristles and eyes

**Figure 2:**
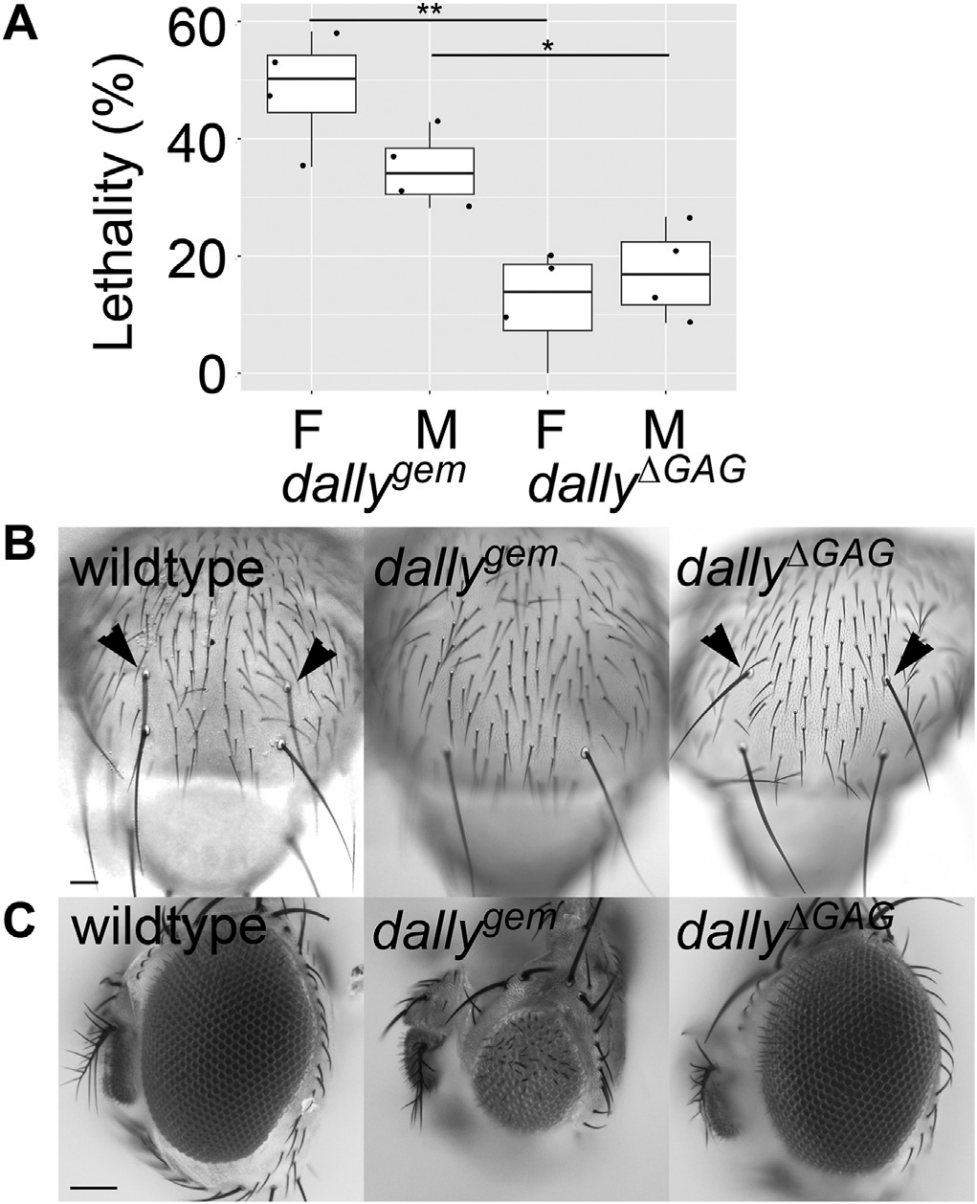
**Lethality and notal bristles of *dally***^**Δ*GAG***^**mutants.***A*, lethality of *dally*^*gem*^ and *dally*^*ΔGAG*^ alleles. The lethality rate of female (F) or male (M) homozygotes for *dally*^*gem*^ and *dally*^*ΔGAG*^ was calculated by four sets of independent experiments. n = 510 for *dally*^*gem*^ females; n = 579 for *dally*^*gem*^ males; n = 1228 for *dally*^*ΔGAG*^ females; n = 1149 for *dally*^*ΔGAG*^ males. Boxes indicate the 25 to 75th percentiles, and the median is marked with a line. The whiskers extend to the highest and lowest values within 1.5 times the interquartile range. *B* and *C*, adult notal bristles and eyes of *dally*Δ*GAG* mutants. Adult notum (*B*) and eyes (*C*) for wildtype (*left*), *dally*^*gem*^ (*middle*), and *dally*^*ΔGAG*^ (*right*) are shown. The penetrance of the loss of anterior dorsocentral bristles and rough eye phenotype in *dally*^*gem*^ is 100% (n = 406 for females and 437 for males). These phenotypes are absent in *dally*^*ΔGAG*^ (penetrance = 0%, n = 852 for females and 812 for males). *Arrowheads* mark anterior dorsocentral bristles. The scale bars represent 250 μm (*B*); 100 μm (*C*). ∗*p* < 0.05; ∗∗*p* < 0.01.

In this study, we used *dally*^*gem*^ as a null allele. *dally*^*gem*^ mutants are semilethal, and approximately half of them die before the adult stage (38). *dally*^*ΔGAG*^ shows a reduced lethality rate (10–20%) (Fig. 2*A*). The milder but non-wildtype phenotypes of *dally*^*ΔGAG*^ are consistent with the idea that the core protein retains coreceptor activity in the absence of HS chains. We found that some *dally* adult phenotypes are absent in *dally*^*ΔGAG*^. For example, *dally* null mutants show the loss of anterior dorsocentral mechanosensory bristles, a specific sensory organ on the notum, with 100% penetrance due to reduced Dpp signaling in the developing notum (Fig. 2*B*, penetrance = 100%, n = 406 for females and 437 for males, (21)). We found that this phenotype completely disappeared in *dally*^*ΔGAG*^ (Fig. 2*B*, penetrance = 0%, n = 852 for females and 812 for males).

Similarly, *dally* null alleles exhibit a fully penetrant rough eye phenotype (38). In contrast, *dally*^*ΔGAG*^ shows normal patterning of the eye (Fig. 2*C*). These observations indicate that HS chains are dispensable for Dally function in notal sensory organ specification and eye morphogenesis.

### *dally*^*ΔGAG*^ shows reduced severity of wing vein V and wing margin patterning defects compared with *dally* null mutants

**Figure 3:**
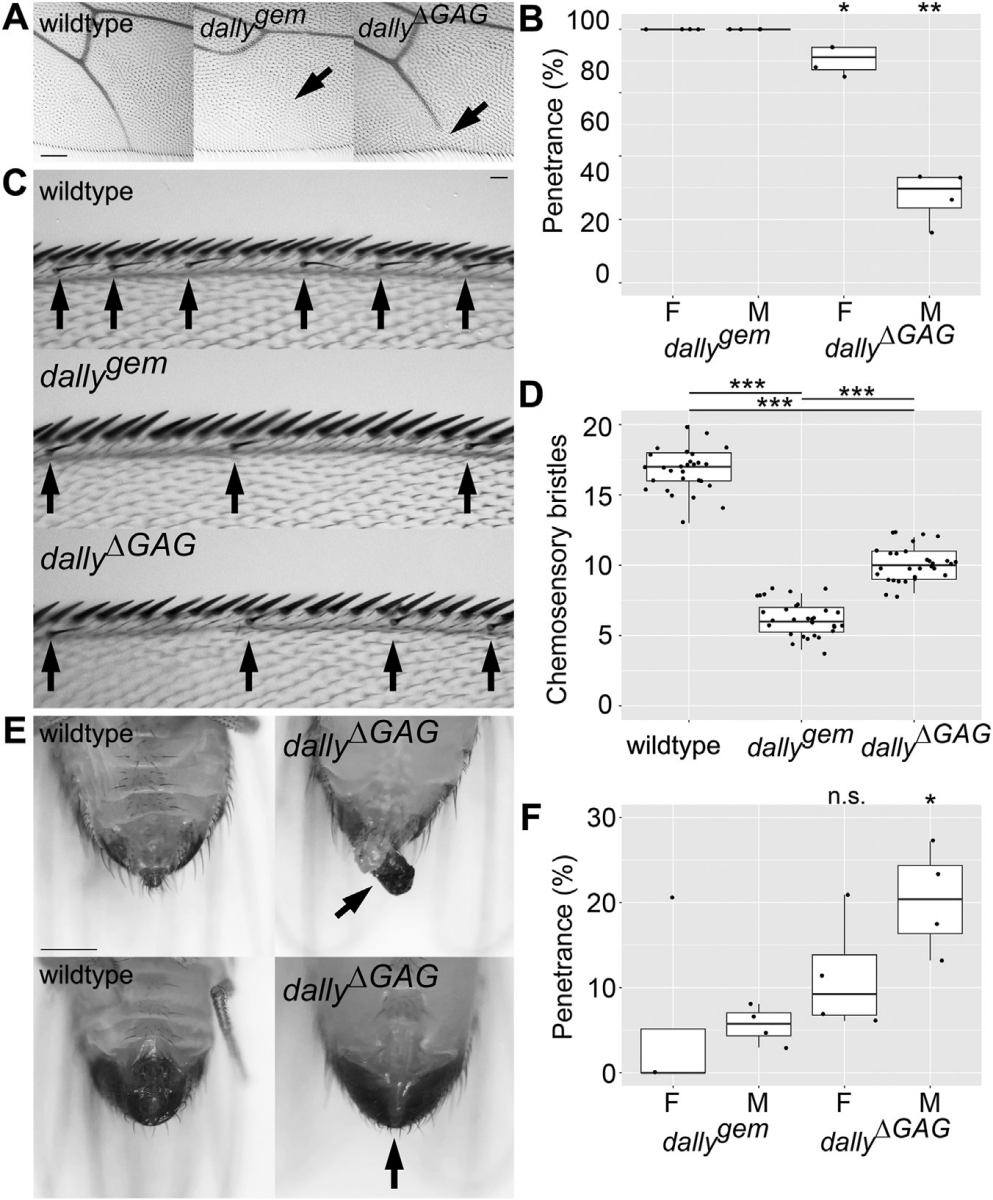
**Analyses of *dally***^**Δ*GAG***^**phenotypes.***A*, posterior portions of wing vein V of adult wings of wildtype (*left*), *dally*^*gem*^ (*middle*), and *dally*^*ΔGAG*^ (*right*) are shown. *B*, the penetrance of the wing vein V defect in females (F) and males (M) of *dally*^*gem*^ and *dally*^*ΔGAG*^. The *box plot* summarizes the penetrance data from four sets of independent experiments. n = 104 for *dally*^*gem*^ females; n = 142 for *dally*^*gem*^ males; n = 376 for *dally*^*ΔGAG*^ females; n = 337 for *dally*^*ΔGAG*^ males. *C*, the anterior wing margin of wildtype (*top*), *dally*^*gem*^ (*middle*), and *dally*^*ΔGAG*^ (*bottom*). Chemosensory bristles are marked by *arrows*. *D*, the quantification of chemosensory bristles. n = 30 for wildtype; n = 30 for *dally*^*gem*^; n = 30 for *dally*^*ΔGAG*^. *E*, genitalia defects of *dally*^*ΔGAG*^. Female (*top*) and male (*bottom*) genitalia are shown for wildtype (*left panels*) and *dally*^*ΔGAG*^ (*right panels*). Arrows show hyperplasia of female genitalia and a complete loss of male genitalia. *F*, the penetrance of the genitalia defects in females (F) and males (M) of *dally*^*gem*^ and *dally*^*ΔGAG*^. The box plot represents the penetrance data from four sets of independent experiments. n = 104 for *dally*^*gem*^ females; n = 142 for *dally*^*gem*^ males; n = 376 for *dally*^*ΔGAG*^ females; n = 337 for *dally*^*ΔGAG*^ males. The scale bars represent 100 μm (*A*); 30 μm (*C*); 200 μm (*E*). ∗*p* < 0.05; ∗∗*p* < 0.01; ∗∗∗*p* < 0.001; n.s., not significant.

Dally coreceptor is required for normal gradient formation of Dpp. In the absence of *dally*, the Dpp signal gradient steeply drops near the source of Dpp (6, 7). As a result, the *dally* mutant wing is narrow in the AP axis and loses a posterior part of the longitudinal wing vein 5 (L5) ((38); Fig. 3*A* middle). The penetrance of this phenotype in *dally*^*gem*^ is 100% for both sexes (Fig. 3*B*). We found that *dally*^Δ*GAG*^ retains this phenotype with much reduced severity and lower penetrance (Fig. 3, *A* right and *B*).

Dally also regulates Wg-dependent specification of the sensory organs at the wing margin. The anterior wing margin bears rows of different types of sensory organs: thick and short mechanosensory bristles at the edge and thinner chemosensory bristles slightly posterior to the edge (39). The number of these bristles is substantially reduced in *dally*^*gem*^ ((21); Fig. 3, *C* and *D*). The bristle formation at the wing margin is partially restored in *dally*^*ΔGAG*^ (Fig. 3, *C* and *D*).

Thus, *dally*^*ΔGAG*^ shows reduced severity of wing vein V and wing margin patterning defects compared with *dally* null mutants. These observations suggest that both the core protein and the HS chains contribute to the Dally activity during the formation of the wing veins and the wing margin.

### *dally*^*ΔGAG*^ shows an increased penetrance of genitalia phenotypes compared with *dally* null mutants

*dally*^*gem*^ males show a complete lack of genitalia, called genitalia-missing, or “gem,” phenotype (38). This phenotype was shown to be a Dpp-dependent specification event (40). The female genitalia are also sometimes reduced, but more often we observe a hyperplasia (Fig. 3*E*). In our culture condition, the penetrance of these genitalia phenotypes in *dally* null mutants are low (<10%, Fig. 3*F*). Interestingly, the penetrance of these phenotypes was modestly increased in *dally*^*ΔGAG*^, both in females and males (Fig. 3*F*). The increase in males but not in females was statistically significant (Fig. 3*F*).

We next asked if expression of wildtype *dally* can rescue the phenotypes of *dally*^*ΔGAG*^. Overexpression of *dally* using a strong driver (*e.g.*, *actin-Gal4*) causes lethality and morphological abnormalities (41). We therefore chose to use a *heatshock (hs)-dally* transgene, which induces a modest level of *dally* expression without producing morphological defects at 29 °C (42). With this condition, expression of wildtype *dally* almost completely rescued the lethality and the adult phenotypes of *dally*^*ΔGAG*^, including wing vein V and genitalia defects (Fig. S2). This finding showed that these phenotypes observed in *dally*^*ΔGAG*^ are ascribed to reduced functioning of Dally.

### *dally*^*ΔGAG*^ shows ectopic wing vein formation

**Figure 4:**
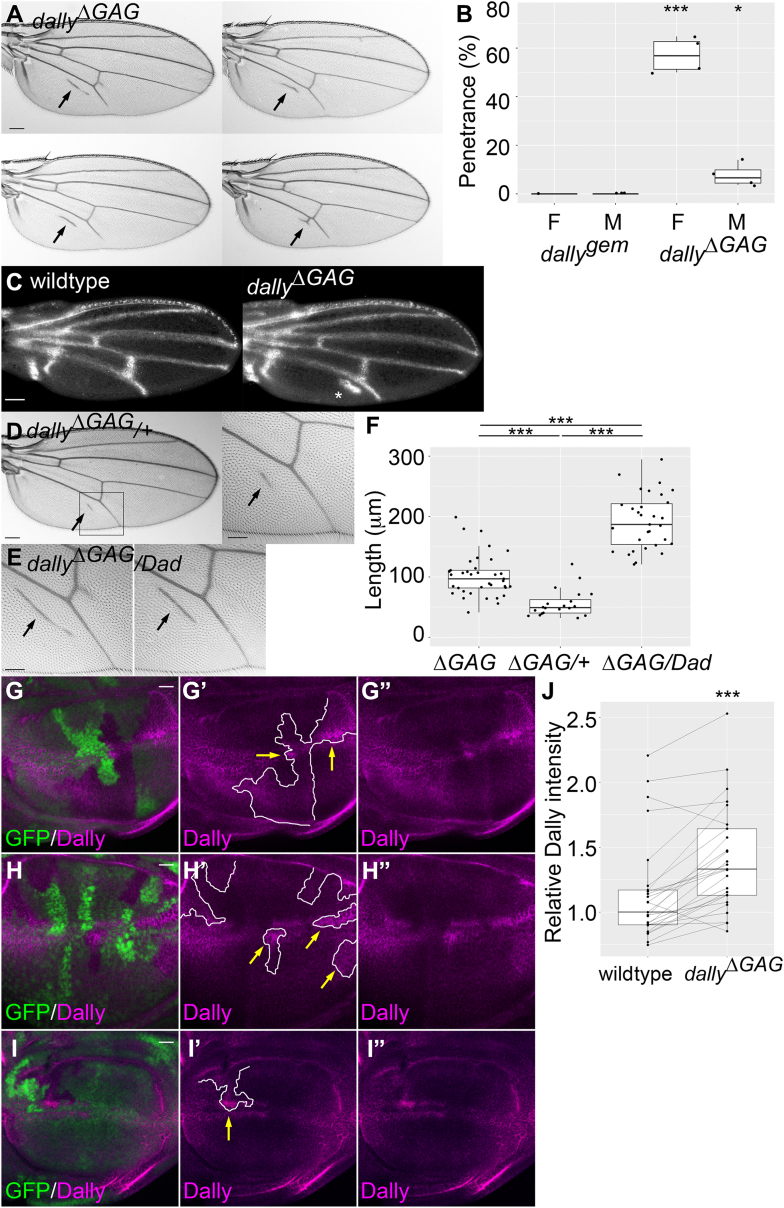
***dally***^**Δ*GAG***^***shows* an ectopic wing vein phenotype.***A*, four examples of adult wings of *dally*^*ΔGAG*^. *dally*^*ΔGAG*^ shows ectopic wing vein formation posterior to wing vein L5. *Arrows* point to the ectopic wing veins. *B*, the penetrance of the posterior wing vein defect. The *box plot* summarizes the penetrance data from four sets of independent experiments. This phenotype has never been observed in *dally*^*gem*^. n = 104 for *dally*^*gem*^ females; n = 142 for *dally*^*gem*^ males; n = 376 for *dally*^*ΔGAG*^ females; n = 337 for *dally*^*ΔGAG*^ males. *C*, pupal wings from wildtype (*left*) and *dally*^*ΔGAG*^ (*right*) stained with anti-pMad antibody. Ectopic pMad signal was detected posterior to the wing vein L5 in *dally*^*ΔGAG*^ pupal wing (*asterisk*, penetrance = 50%, n = 10 for wildtype; n = 10 for *dally*^*ΔGAG*^). *D*, the posterior wing vein defect in *dally*^*ΔGAG*^ heterozygotes (*dally*^*ΔGAG*^/+). The magnified image on the *right* is from the outlined inset from the *left* image. *E*, *Dad*, the inhibitory Smad, enhances the posterior wing vein defect of *dally*^*ΔGAG*^ heterozygotes. Arrows indicate posterior ectopic veins. *F*, the quantification of posterior ectopic vein length for *dally*^*ΔGAG*^ homozygotes (*ΔGAG*), *dally*^*ΔGAG*^/+ (*ΔGAG*/+), and *dally*^*ΔGAG*^/*Dad* (*ΔGAG*/*Dad*). n = 37 for *dally*^*ΔGAG*^ homozygotes; n = 20 for *dally*^*ΔGAG*^/+; n = 31 for *dally*^*ΔGAG*^/*Dad*. *G*–*I"*, three examples are shown for wing discs with *dally*^*ΔGAG*^ homozygous mutant clones stained with anti-Dally antibody. The loss of GFP signal marks the mutant clones (*G*–*I*). Anti-Dally antibody signals are shown in magenta. The clone borders are marked with white lines (*G′*–*I*′). Areas of *dally*^*ΔGAG*^ clones with particularly high levels of anti-Dally staining are marked with yellow arrows (*G*′–*I*′). *J*, quantification of anti-Dally antibody staining intensity in *dally*^*ΔGAG*^ mutant clones. Anti-Dally antibody signal intensity in randomly selected *dally*^*ΔGAG*^ mutant clones was compared with that in immediate neighboring wildtype cells (n = 25 pairs). Of 25 *dally*^*ΔGAG*^ mutant clones, 23 showed the elevation of Dally signal intensity compared with that in neighboring wildtype cells. Of 17 wing discs, 15 bore *dally*^*ΔGAG*^ mutant clones with this effect. The scale bars represent 200 μm (*A*, *D left side*); 100 μm (*C*, *D right side*, *E*); 20 μm (*G*–*I’’*). ∗*p* < 0.05; ∗∗∗*p* < 0.001.

Unexpectedly, we found a new, *dally*^*ΔGAG*^-specific phenotype, which has never been observed in the null mutants. *dally*^*ΔGAG*^ shows ectopic wing vein formation at a specific region of the wing, which is posterior to the most posterior wing vein, L5 (Fig. 4*A*). The penetrance of this phenotype is approximately 60% in females (Fig. 4*B*).

Ectopic vein structures can be induced by ectopic activation of Dpp/BMP signaling. To examine the patterns of Dpp activity in developing *dally*^*ΔGAG*^ wings, we stained *dally*^*ΔGAG*^ pupal wing with anti-phosphorylated Mad (pMad) antibody at 24 h after puparium formation. At this stage, pMad staining mainly marks future longitudinal veins and crossveins (Fig. 4*C*). In the *dally*^*ΔGAG*^ pupal wing, ectopic pMad signal was detected posterior to wing vein L5 (Fig. 4*C*), confirming that the ectopic vein phenotype in *dally*^*ΔGAG*^ is associated with ectopic Dpp signaling.

Interestingly, we found that this phenotype is semidominant. *dally*^*ΔGAG*^/+ heterozygotes show a smaller ectopic vein at the same location (Fig. 4, *D* and *F*). Thus, the formation of this posterior ectopic vein is *dally*^*ΔGAG*^ dosage dependent. The small ectopic vein reflects a delicate balance of ectopic Dpp signaling, and it is sensitive to a further change in the signaling dosage. For example, we found that this phenotype is sensitive to *Dad*. *Dad* is the *Drosophila* ortholog of inhibitory Smads. Vertebrate inhibitory Smads negatively regulates signaling of TGF-β family ligands by inhibiting phosphorylation of R-Smads (43, 44, 45). In *Drosophila*, heterozygosity of *Dad* slightly increases Dpp signaling but does not show any defect by itself (46, 47). However, it significantly enhances the ectopic vein phenotype of *dally*^*ΔGAG*^ (Fig. 4, *E* and *F*).

These results suggest that Dpp signaling is ectopically activated to induce extra venation in a region of a *dally*^*ΔGAG*^ wing where its ligand concentration is very low. We next asked if the level of Dally core protein is altered in *dally*^*ΔGAG*^ mutant cells. *dally*^*ΔGAG*^ homozygous clones were induced in the wing disc, and Dally core-protein expression was examined using anti-Dally antibody. We found a higher level of Dally core protein in *dally*^*ΔGAG*^ mutant cells compared with surrounding *dally*^*ΔGAG*^/+ and +/+ cells (Fig. 4, *G*–*I*"). Quantification of anti-Dally antibody signal inside and outside of *dally*^*ΔGAG*^ mutant clones is shown in Figure 4*J*.

### Most *dlp* mutant phenotypes are absent in *dlp*^*ΔGAG*^ allele

Unlike *dally*^*ΔGAG*^, the *dlp*^*ΔGAG*^ allele is surprisingly healthy. *dlp*^*ΔGAG*^ homozygotes are viable and fertile and show no obvious morphological defect. Given that *dlp* null is a very sick mutant with 100% lethality at larval stage, this difference was striking.

**Figure 5:**
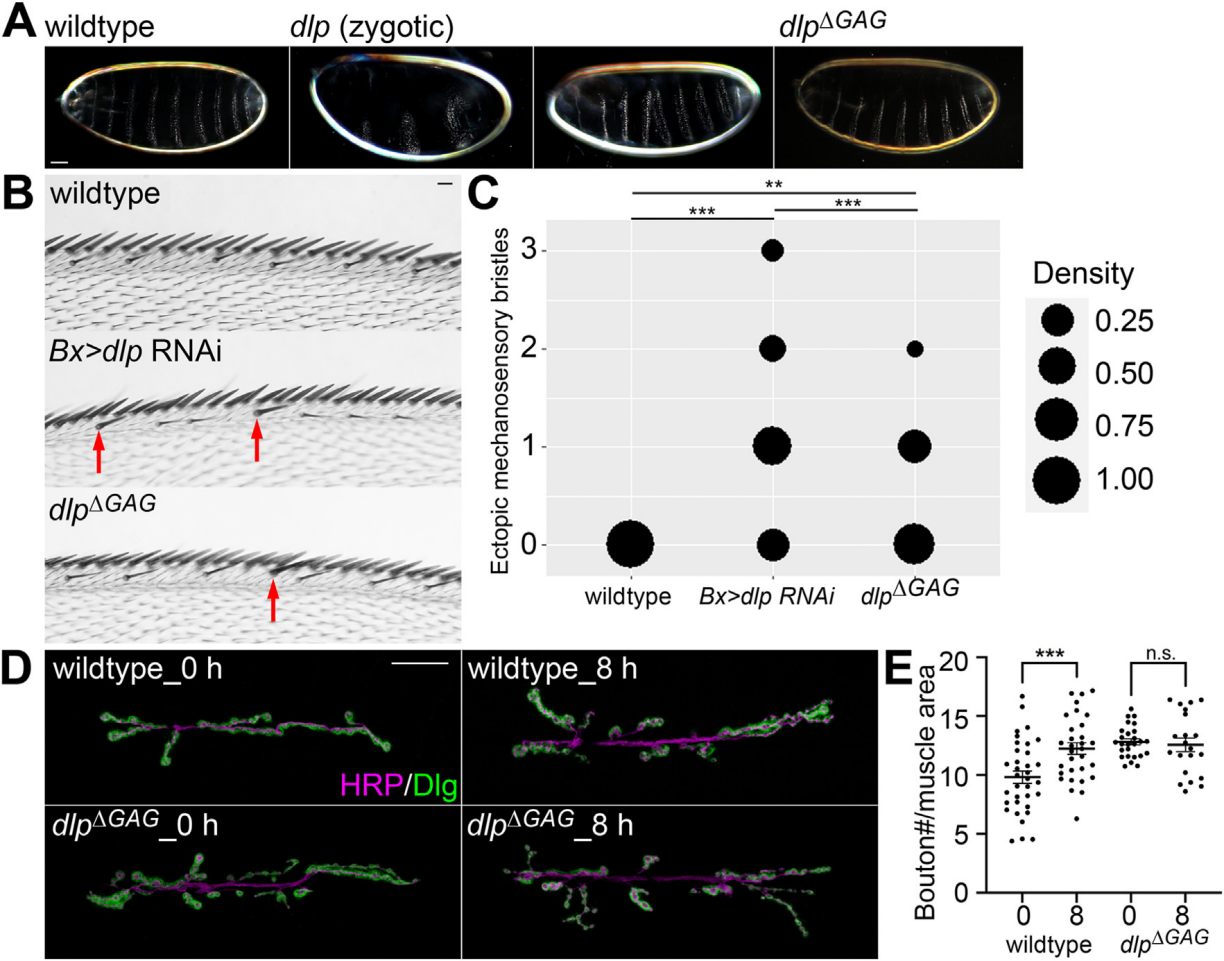
**Analyses of *dlp***^**Δ*GAG***^**phenotypes.***A*, embryonic cuticle is shown for wildtype, *dlp*^*MH20*^ (zygotic), and *dlp*^*ΔGAG*^. Two examples are shown for *dlp*^*MH20*^ mutants, which show a segment-polarity phenotype. *B*, ectopic mechanosensory bristles at the anterior wing margin in the wing of wildtype (*top*), *Bx>dlp RNAi* (*middle*), and *dlp*^*ΔGAG*^ (*bottom*). *Red arrows* show the ectopic bristles. *C*, density plot showing the ratio of animals with the indicated number of the ectopic mechanosensory bristles for each genotype. n = 30 for wildtype; n = 29 for *Bx>dlp RNAi*; n = 30 for *dlp*^*ΔGAG*^. *D*, neuromuscular junction was stained with anti-HRP (axon) and anti-Dlg (postsynapse) antibodies. Signals for HRP and Dlg are shown in magenta and green, respectively. *E*, quantification of bouton number per muscle area (10,000 μm^2^) in the neuromuscular junction of wildtype and *dlp*^*ΔGAG*^ at 0 or 8 h of starvation. n = 35 for wildtype hour 0; n = 31 for wildtype hour 8; n = 26 for *dlp*^*ΔGAG*^ hour 0; n = 21 for *dlp*^*ΔGAG*^ hour 8. The scale bars represent 50 μm (*A*); 30 μm (*B*); 25 μm (*D*). ∗∗*p* < 0.01; ∗∗∗*p* < 0.001; n.s. not significant.

Dlp is a Hh coreceptor, and the *dlp* mutant embryos show a segment polarity phenotype ((12); Fig. 5*A*). The segment polarity phenotype of *dlp*^*MH20*^ embryos shown in Figure 5*A* are relatively mild because they are only zygotically null and rescued by maternal supply. *dlp*^*ΔGAG*^ embryos do not show this phenotype despite the fact that these embryos are both maternally and zygotically homozygous with no rescue by wildtype *dlp* mRNA (Fig. 5*A*).

Dlp is known to show a biphasic effect on Wg signaling: it downregulates Wg signaling near the Wg-expressing cells but upregulates it where the ligand concentration is low (17, 36, 48, 49, 50). Therefore, *dlp* knockdown shows ectopic mechanosensory bristles, slightly shifted from the edge due to increased Wg signaling at the wing margin, which is formed by high levels of Wg signaling (50); Fig. 5*B*). We observed a mild phenotype of the ectopic mechanosensory bristles in *dlp*^*ΔGAG*^, with a significantly lower expressivity than *Bx>RNAi* knockdown animals (Fig. 5, *B* and *C*).

### *dlp*^Δ*GAG*^ shows neuromuscular junction phenotypes

We previously showed that the *trans* coreceptor activity of glypicans needs GAG chains *in vitro* (10). Dlp functions in *trans*, with the most obvious example being its role at the neuromuscular junction (NMJ) during larval development. At the NMJ, Lar, a protein tyrosine phosphatase, functions on the presynaptic membrane to promote bouton formation (51). Two types of HSPGs on the postsynaptic membrane regulate Lar signaling in an opposing manner: Syndecan promotes Lar activity and Dlp suppresses it (52). Therefore, *dlp* mutants show an increased number of boutons (Fig. 5, *D* and *E*; (53)). In addition to the control of Lar activity during development, Dlp is required for starvation-induced synaptic plasticity. Under starvation conditions, larvae show increases in locomotor activity and the number of synaptic boutons at the NMJ. Such increases in locomotor speed and bouton production depend on noncanonical BMP signaling (Fig. 5*E*; (53)).

We found that *dlp*^*ΔGAG*^ failed to suppress bouton formation under normal conditions (Fig. 5, *D* and *E*). This is the same phenotype as *dlp* knockdown animals we have previously shown (53). Statistical analyses revealed no significant difference in the bouton numbers per area between *dlp*^*ΔGAG*^ and *dlp* knockdown animals (Fig. S3). Furthermore, *dlp*^*ΔGAG*^ failed to induce starvation-induced synaptic plasticity, resulting in the same number of boutons before and after 8-h food deprivation (Fig. 5*E*), which is also consistent with *dlp* knockdown (Fig. S3; (53)). These results indicated that HS chains of Dlp are indispensable at least for its NMJ functions.

### *dlp*^*ΔGAG*^ is lethal in the absence of *dally*

**Figure 6:**
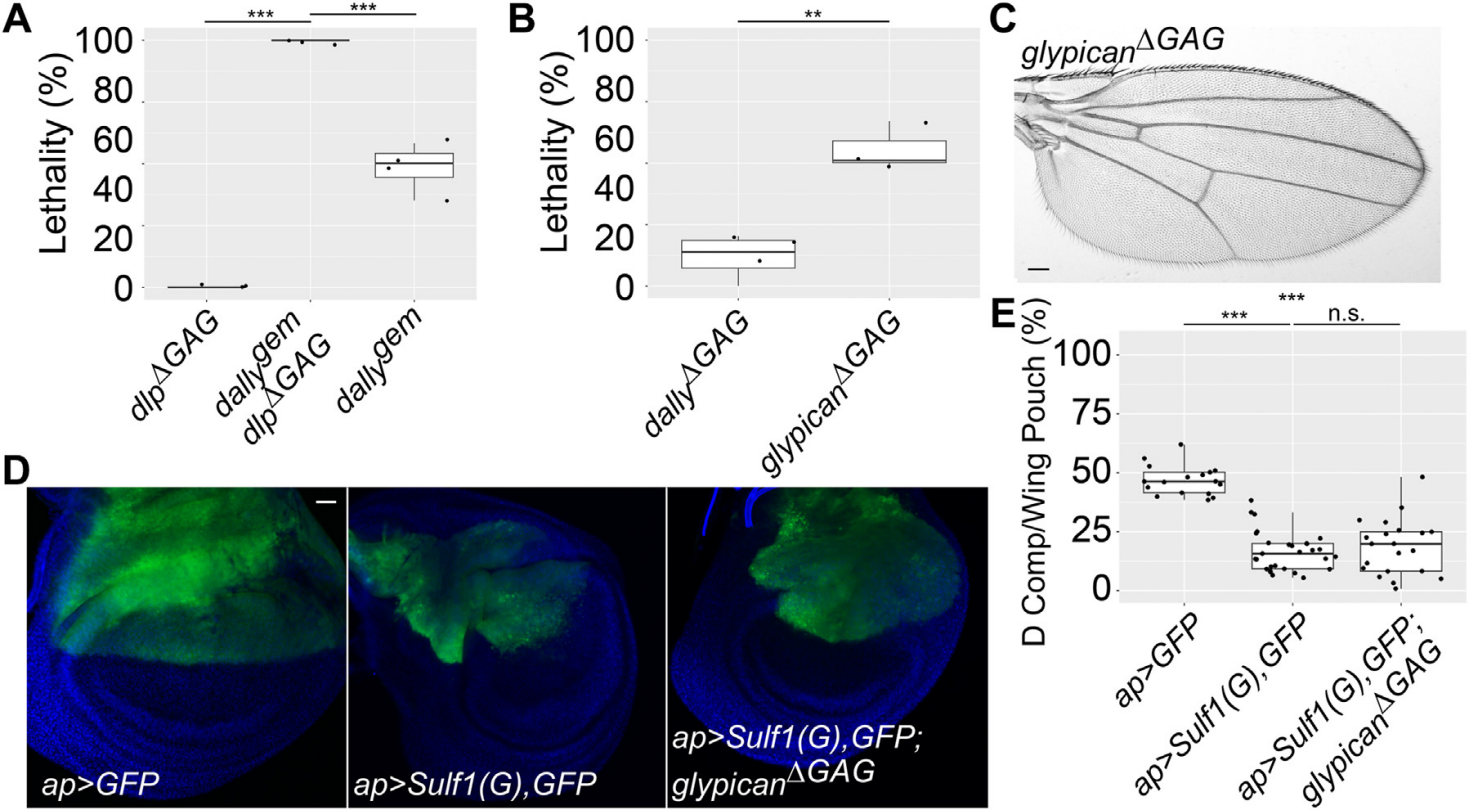
**Analyses of *dally***^***gem***^***dlp***^***ΔGAG***^**and *dally***^***ΔGAG***^***dlp***^***ΔGAG***^**double mutants.***A*, lethality of *dlp*^*ΔGAG*^, *dally*^*gem*^*dlp*^*ΔGAG*^, and *dally*^*gem*^ alleles. *B*, lethality of *dally*^*ΔGAG*^ and *dally*^*ΔGAG*^*dlp*^*ΔGAG*^ (*glypican*^*ΔGAG*^) alleles. For (*A* and *B*), the lethality rate of females was calculated by three to four sets of independent experiments. n = 497 for *dlp*^*ΔGAG*^; n = 140 for *dally*^*gem*^*dlp*^*ΔGAG*^; n = 406 for *dally*^*gem*^; n = 852 for *dally*^*ΔGAG*^; n = 366 for *glypican*^*ΔGAG*^. *C*, an example of an adult *glypican*^*ΔGAG*^ wing. *D*, wing discs are shown for *ap-Gal4/UAS-GFP* (*ap>GFP*; *left*), *ap-Gal4/UAS-Sulf1(G) UAS-GFP* (*ap>Sulf1(G), GFP*; *middle*), and *ap-Gal4/UAS-Sulf1(G) UAS-GFP; glypican*^*ΔGAG*^ (*ap>Sulf1(G), GFP; glypican*^*ΔGAG*^; *right*). *E*, the percentage of the dorsal compartment area within the wing pouch was quantified for each genotype. n = 17 for *ap>GFP*; n = 29 for *ap>Sulf1(G), GFP*; n = 21 for *ap>Sulf1(G), GFP; glypican*^*ΔGAG*^. The scale bars represent 200 μm (*C*); 20 μm (*D*). ∗∗*p* < 0.01; ∗∗∗*p* < 0.001; n.s. not significant.

Although we found important roles of Dlp HS chains at the NMJ, overall phenotypes of *dlp*^*ΔGAG*^ are surprisingly mild. This prompted us to analyze whether Dally plays a role in this allele. To address this question, we generated the *dally*^*gem*^ *dlp*^*ΔGAG*^ double mutant. Remarkably, the double mutant showed 100% lethality (Fig. 6*A*). This suggests that most functions of Dlp HS chains can be complemented by Dally, except for their roles at the NMJ.

The lethality data also showed that *dally*^*gem*^ *dlp*^*ΔGAG*^ double mutants show higher levels of lethality than *dally*^*gem*^ single mutants (Fig. 6*A*), indicating that Dlp HS chains function to support the survival of *dally* mutants. Thus, although previous studies indicated that Dally and Dlp have only limited functional redundancy in the wildtype background, they show close functional relationships when one of the glypicans is disrupted.

### The phenotypes of *glypican*^*ΔGAG*^

We next recombined *dally*^*ΔGAG*^ and *dlp*^*ΔGAG*^ to generate the double mutant, or *glypican*^*ΔGAG*^. Based on the lethality data, they are more severe than *dally*^*ΔGAG*^ (Fig. 6*B*), suggesting a partial functional redundancy between Dally and Dlp HS chains. Interestingly, although *glypican*^*ΔGAG*^ is homozygous for *dally*^*ΔGAG*^, they do not show the posterior ectopic wing vein (Fig. 6*C*). Thus, Dlp HS chains not only weaken *dally* mutant phenotypes but also are required for the formation of the posterior ectopic wing vein in *dally*^*ΔGAG*^.

Numerous previous studies have established the coreceptor functions of glypicans in morphogen signaling (9). On the other hand, only limited cases are known for nonglypican HSPGs as a coreceptor (9). It is worth noting that there is a large gap between the phenotypes of *glypican*^*ΔGAG*^ and the HS biosynthetic gene mutants, such as *ttv* and *sfl*. Zygotic null mutants for these biosynthetic genes show 100% lethality during larval to pupal stages (28, 29, 30), while 40% of *glypican*^*ΔGAG*^ mutants survive to the adult stages (Fig. 6*B*). This difference in the lethality levels between *ttv* (or *sfl*) and *glypican*^*ΔGAG*^ suggested that nonglypican HSPGs play a role in morphogen signaling. To confirm this idea, we tested the effect of *Sulf1* overexpression in the *glypican*^*ΔGAG*^ background. *Sulf1* inhibits multiple morphogen signaling pathways, including the Wg, Hh, and Dpp pathways (22, 23). Overexpression of a Golgi-tethered form of *Sulf1* (*Sulf1-Golgi*, or *Sulf1(G)*) in the developing wing by *ap-Gal4*, a dorsal compartment-specific Gal4 driver, decreases the proliferation of dorsal cells in a cell-autonomous manner (22). This results in a significant reduction in the dorsal cell/entire wing pouch area ratio (Fig. 6, *D* and *E*). A similar effect of *Sulf1(G)* expression was observed in *glypican*^*ΔGAG*^ homozygous wing discs (Fig. 6, *D* and *E*). This confirmed that morphogen signaling is mediated by HS chains on nonglypican HSPGs when glypican HS modification is impaired.

**Figure 7:**
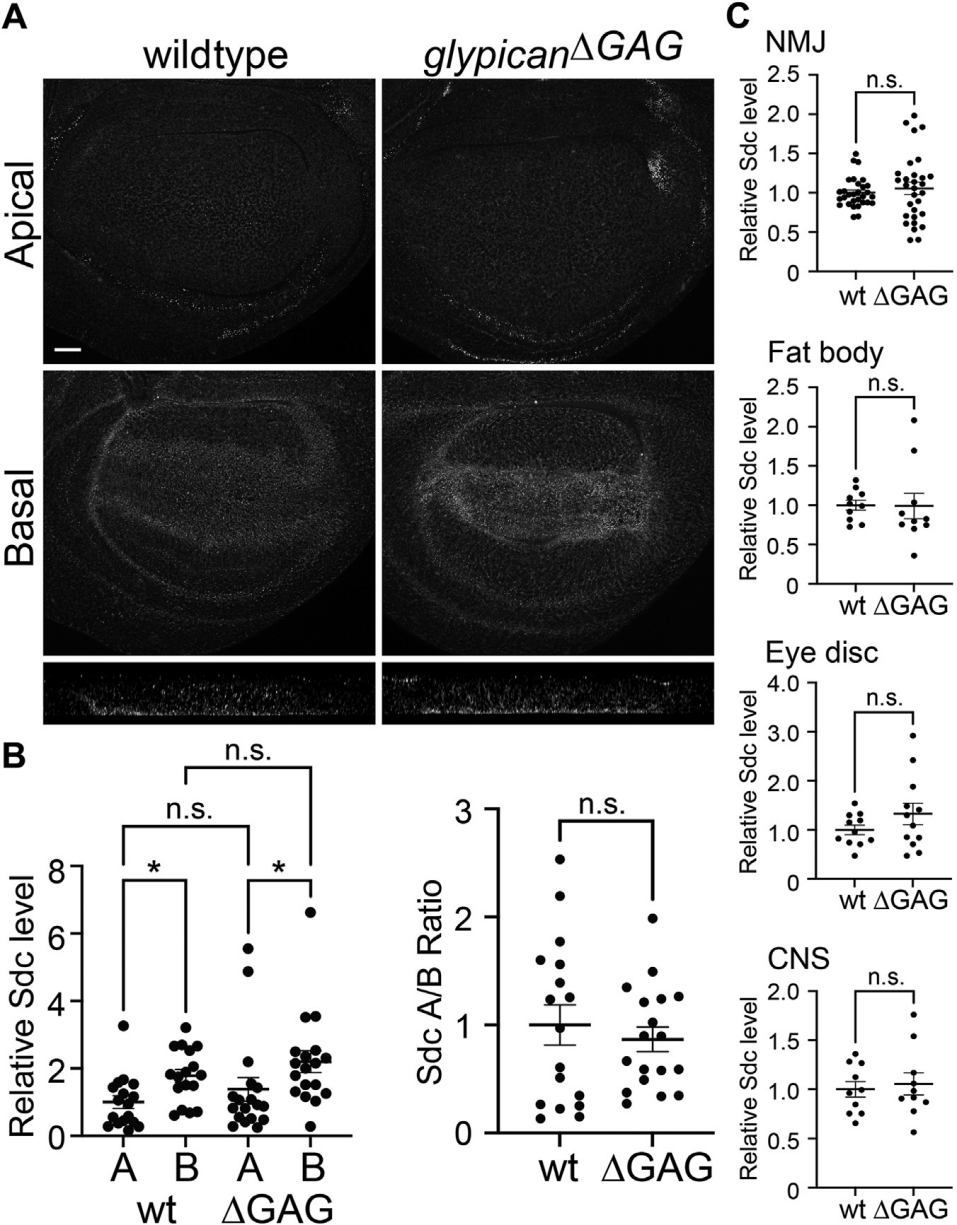
**Sdc expression in *glypican***^***ΔGAG***^**.***A*, wing discs from wildtype (*left*) and *glypican*^*ΔGAG*^ homozygotes (*right*) stained with anti-Sdc antibody. Apical (*top*) and basal (*middle*) sections are shown. The bottom pales show Z-sections near the dorsoventral boundary (*apical/top-basal/bottom*). *B*, quantification of anti-Sdc antibody signal intensity. Relative signal intensity in the apical (*A*) and basal (*B*) sections are compared between wildtype (wt) and *glypican*^*ΔGAG*^ homozygotes (ΔGAG). The ratio of apical and basal signal intensity is also compared (*right*). *C*, quantification of anti-Sdc antibody signal intensity in other tissues (neuromuscular junction [NMJ], fat body, eye disc, and central nervous system [CNS]) from wildtype (wt) and *glypican*^*ΔGAG*^ homozygotes (ΔGAG). The scale bar represents 20 μm (*A*). ∗*p* < 0.05; n.s., not significant.

Previous studies have shown that a disruption of HS functions is compensated by various feedback systems, suggesting a possibility that such compensation systems function in ΔGAG alleles. For example, upregulation of nonglypican HSPGs may contribute to the mild phenotypes of the ΔGAG alleles. Therefore, we asked if Syndecan (Sdc) expression is altered in *glypican*^*ΔGAG*^. Anti-Sdc antibody staining of wing disc, NMJ, fat body, eye disc, and central nervous system showed no significant difference in Sdc levels and distribution between wildtype and *glypican*^*ΔGAG*^ (Fig. 7). Thus, different mechanisms (*e.g.*, other proteoglycans, HS sulfation) may be involved in ΔGAG mutants.

## Discussion

Using glypican "ΔGAG" alleles in which all endogenous GAG-attachment serine residues are replaced with alanine residues, we found that levels of HS dependency of the two *Drosophila* glypicans, Dally and Dlp, are strikingly different. Regarding *dally* alleles, the *dally*^*ΔGAG*^ allele generally shows less severe phenotypes compared with *dally* null mutants. We classified *dally* null mutant phenotypes into two classes: (1) HS chains being dispensable, which includes notal sensory bristle formation and eye morphogenesis, and (2) both HS chains and core protein being required for proper development, which includes the formation of wing vein V, wing margin sensory bristles, and genitalia. The defective formation of anterior dorsocentral bristles, wing vein V, and genitalia in *dally* mutants is caused by reduced Dpp signaling. Therefore, the two classes were not simply dependent on the morphogen pathway that *dally* regulates but are likely to be context dependent.

In contrast to *dally*^*ΔGAG*^, we observed only limited morphological defects in *dlp*^*ΔGAG*^. This is a striking contrast to the zygotic null mutants of *dlp*, which are 100% lethal at larval stage (both maternal and zygotic null is 100% lethal at embryonic stage). We found that the *dlp*^*ΔGAG*^ allele is healthy only due to the presence of Dally, as it is 100% lethal in the *dally* null background.

Although HS chains of Dlp are dispensable for most Dlp functions, analyses of the double mutants, *dally*^*gem*^ *dlp*^*ΔGAG*^ and *dally*^*ΔGAG*^ *dlp*^*ΔGAG*^ (*glypican*^*ΔGAG*^), revealed additional activities of Dlp HS chains in these mutant backgrounds. They show the ability to (1) weaken *dally* mutant phenotypes, (2) complement the loss of Dally HS chains, and (3) contribute to the formation of the posterior ectopic wing vein in *dally*^*ΔGAG*^. Although there is very little functional redundancy between Dally and Dlp under normal environmental conditions, genetic manipulations inducing partially truncated coreceptor functions revealed intimate relationships between the glypicans.

It has been believed that the glypicans function as coreceptors for most morphogen ligands in *Drosophila*. When glypican HS modification is impaired, however, nonglypican HSPGs are able to function in morphogen signaling (Fig. 6, *D* and *E*). As disruptions of HS functions are known to trigger various compensation systems to minimize their impact, it is possible that the mild phenotypes of the ΔGAG alleles are due to a compensatory feedback response. Possible mechanisms of such feedback mechanisms include, but are not limited to, the regulation of HSPG core-protein expression and/or distribution, HS sulfation, and HS chain length. No significant change in Sdc levels and distribution was observed in *glypican*^*ΔGAG*^ (Fig. 7). This observation suggests that different mechanisms may contribute to the modest ΔGAG mutant phenotypes. Further studies are required to test this possibility and to determine the molecular nature of the unexpectedly mild phenotypes of the ΔGAG mutants.

In addition to known *dally* mutant phenotypes, *dally*^*ΔGAG*^ shows the formation of an ectopic vein in the posterior region of the wing. This region of the pupal wing exhibits ectopic pMad, and the phenotype is enhanced by *Dad*. These findings suggest that Dpp signaling is ectopically activated to induce extra venation in a region where the ligand concentration is very low. It is unknown how this morphogen signaling is increased by the loss of Dally HS chains, but there are a few possibilities. First, the ligand gradient may be altered. Unlike *dally* null mutants, in which the Dpp gradient quickly drops near the source of Dpp (6, 7), the gradient may be expanded in *dally*^*ΔGAG*^ as Dally^ΔGAG^ should have lower affinity, or be less “sticky,” to Dpp. Therefore, the cells at the periphery may receive a higher level of Dpp, which can specify an ectopic vein fate. Second, as shown in Figure 4, *G*–*I*", *dally*^*ΔGAG*^ mutant cells have an increased level of Dally core protein. Pentagone is a secreted factor that regulates the Dpp gradient by promoting Dally internalization and degradation (54). Recently, another soluble factor, Nord, was found to show a similar activity (55). Both proteins are heparin-binding factors and thus are likely to bind Dally through HS chains. If so, Dally^ΔGAG^ may escape from Pent- and Nord-dependent degradation. It is also possible that the *dally* gene is transcriptionally upregulated in *dally*^*ΔGAG*^ mutant cells. Thus, either altered local Dpp concentration or elevated levels of Dally core protein, or both as a combination, may lead to ectopic activation of Dpp signaling.

In the past studies addressing the relative contribution of the protein *versus* sugar moieties to the PG functions using conventional "ΔGAG" overexpression constructs, we tended to assume that "core-protein activity plus GAG activity equals PG activity" as the basis of the assessment. Our study suggests that this idea may be oversimplified. As HSPGs regulate both the signal reception on the cell surface (a cell autonomous function) and ligand distribution in a tissue (a nonautonomous function) (6), the consequence of a *ΔGAG* mutation is regionally different even in the same tissue. For example, *dally*^*ΔGAG*^ shows multiple phenotypes caused by reduced Dpp-Dally signaling (*e.g.*, wing vein V and genitalia defects) and also posterior wing vein phenotype caused by ectopic Dpp signaling. Interestingly, *hs-dally* did not enhance the ectopic wing vein phenotype of *dally*^*ΔGAG*^ but partially suppressed it (Fig. S2*C*). This finding suggests that this phenotype is produced by a combined effect of cell autonomous and nonautonomous consequences from the lack of HS chains. Furthermore, it is worth noting that *glypican*^*ΔGAG*^ provides a unique cellular condition: there are no HS attachment sites on the glypicans, which normally function as coreceptors for most morphogen signaling, but the HS biosynthetic machinery is intact. Altogether, *ΔGAG* mutants will be highly useful to address important biological questions, such as molecular mechanisms of morphogen gradient formation and scaling.

## Experimental procedures

### *Drosophila* strains

The following fly strains were used in this study: Oregon-R, *Dad*^*p1883Δ32*^ (56), *dally*^*gem*^ (38, 40), *hs-dally* (42), *dlp*^*MH20*^, a null allele for *dlp* (17), *Bx-GAL4* (BDSC #8860), *ap-Gal4*, *UAS-GFP* (BDSC #1521), *UAS-Sulf1(G)* (22), and *UAS-dlp RNAi*^*HMS00875*^, a UAS short-hairpin RNAi strain for *dlp* (57). Flies were raised on a standard cornmeal fly medium at 25 °C unless otherwise indicated.

### Generation of *dally*^*ΔGAG*^ and *dlp*^*ΔGAG*^ mutant alleles

In order to generate *dally*^*ΔGAG*^, five GAG-attachment serine residues at positions 549, 569, 573, 597, and 601 of the *dally* cDNA were substituted with alanine residues *via* CRISPR/Cas9-mediated gene editing by injecting a donor DNA construct and a *dally* sgRNA.

To construct *pHD-dally*^*ΔGAG*^ donor DNA, a part of the *dally* coding sequence containing 361-bp gBlock carrying *dally*^*ΔGAG*^ mutations (synthesized by IDT) were cloned into the pHD-DsRed-attP backbone using NEBuilder HiFi DNA Assembly Master Mix (E2611S, New England Biolabs). To construct a *dally* sgRNA clone, two primers, 5′-CTTCGTGTTCAATCCC TTGGGCTG-3′ and 5′-AAACCAGCCCAAGGGATTGAACAC-3′, were annealed and cloned into the BbsI site of *pU6-BbsI-chiRNA* plasmid.

To generate *pHD-dlp*^*ΔGAG*^ donor DNA, similar to *dally*^*ΔGAG*^, we replaced five GAG-attachment serine residues, at positions 625, 629, 631, 643, and 683, of the *dlp* cDNA with alanine residues. This sequence was cloned into the pHD-DsRed-attP backbone. To generate a *dlp* sgRNA clone, two primers, 5′-TTCGTGGAGCTGGTTCTGGATCC-3′ and 5′-AACGGATCCAGAACCAGCTCCAC-3′, were annealed and cloned into the BbsI site of *pU6-BbsI-chiRNA* plasmid.

DNA mixture containing donor DNA plasmid (250 ng/μl) and sgRNA (50 ng/μl) were injected by GenetiVision into the *nos-Cas*9 embryos (*yw*; *nos-Cas9(y+)/CyO*) for *dally*^*ΔGAG*^ mutant, and the *vasa-Cas*9 embryos (BDSC #51323) for *dlp*^*ΔGAG*^ mutant. The mutant fly lines were screened by PCR, and the genomic DNA sequences were molecularly confirmed by Sanger DNA sequencing. The obtained alleles were backcrossed to Oregon-R for six generations.

### Preparation of embryonic cuticles, adult wings, nota, and eyes

The standard embryo collection for preparation of cuticles consisted of a 4-h egg collection, followed by incubation for 20 h at 25 °C (Tsuda *et al*., 1999). Embryos were fixed, and devitellinized cuticles were prepared with standard procedures.

The right wings from female flies were dehydrated in ethanol and subsequently with xylene (21, 58). Adult cuticles of the notum were boiled in 2.5 N sodium hydroxide, washed in distilled water, and dehydrated in 2-propanol. The specimens were mounted in Canada balsam (Benz Microscope, BB0020).

Left eyes from adult males were imaged using a Nikon AZ100M microscope. Z-stacks were captured for each eye and compiled into a single focused image using NIS-Elements.

### Immunohistochemistry and immunoblot analysis

Immunostaining of the wing discs and ovaries was performed as described (58, 59, 60). The primary antibodies used were as follows: Alexa Fluor 647–conjugated AffiniPure Goat anti-HRP (1:200, Jackson ImmunoResearch), mouse anti-Discs large (Dlg, 4F3) (1:20, Developmental Studies Hybridoma Bank [DSHB]), rabbit anti-pSmad3 (1:1000, Epitomics, 1880-1), and rabbit anti-Sdc (1:250, (52)). Images were obtained using a Zeiss 710 laser scanning confocal microscope.

Anti-Dally polyclonal antibody was raised using a synthetic peptide, DSRAKDAVGGSTHQC (Biologica Co). For immunoblot analysis, protein samples were extracted from *Drosophila* adult whole body by SDS sample buffer. The peptide was injected into guinea pig, and the antibody was affinity purified. Mouse anti-Dlp (1:200, DSHB), guinea pig anti-Dally antibody (1:1000), and mouse anti-α-Tubulin (1:2,000, Sigma-Aldrich, DM1A) were used as primary antibodies. Signals were detected using HRP-conjugated secondary antibodies and Pierce ECL Western Blotting Substrate (Thermo Scientific).

### NMJ analysis

Quantification of synaptic boutons of larvae was performed as described (53). Larval body wall muscles were dissected in Ca^2+^-free HL3 saline (70 mM NaCl, 5 mM KCl, 20 mM MgCl_2_, 10 mM NaHCO_3_, 5 mM trehalose, 115 mM sucrose, and 5 mM Hepes, pH 7.2) and fixed in Bouin's fixative (Sigma-Aldrich) at room temperature for 20 min. Tissues were incubated at 4 °C overnight in primary antibody solutions and then at room temperature for 2 h in secondary antibody solutions. Alexa Fluor 647–conjugated AffiniPure Goat Anti-HRP and mouse anti-Dlg were used as the primary antibodies. The number of type I boutons, defined as axonal swelling, at muscles 6 and 7 in abdominal segment A3 were scored. The image of corresponding muscle was obtained and muscle surface area was calculated by ImageJ software. Bouton number was then normalized to muscle area. For starvation assay, we used larvae showing vigorous feeding in the food slurry that did not climb out of the food. After washing with water using a paintbrush, larvae were placed on 0.7% agar in vials and were left there for 8 h before dissection.

## Data availability

All data are contained within the article.

## Supporting information

This article contains supporting information (53).

## Conflict of interest

The authors declare that they have no conflicts of interest with the contents of this article.
