# Supplementary material for: Differential heparan sulfate dependency of the Drosophila glypicans
Source: J Biol Chem. 2023 Dec 10;300(1):105544. doi: 10.1016/j.jbc.2023.105544 (PMC10796981; doi:10.1016/j.jbc.2023.105544)
Supplement: Supplementary material [file mmc1.docx]

Dally[deltaGAG]

MAARSVRLAQLLLFTLLCGFVGLSAAKHLDLDGIHHHQHHLHSATTHHRRRLQRDSRAKDAVGGSTHQCDAVKSYFESIDIKSSGTYSEKGAICGGNCCNNATELELRDKAAGMFEQLLHHHTSSLRGVLETNAKQFQSHVLELAQISENMTHSLFSKVYTRMVPSSRMMIHQLYTEIMNHLIYTSNYTNSNGQLGRRGIGSVQSNLEEAVRHFFVQLFPVAYHQMVHLSKNNLGDLHEDYVNCLQHNFDEMHPFGDIPQQVQSNLGKSVHMSNVFMNALLQAAEVLSEADALYGEQLTDTCKLHLLKMHYCPNCNGHHSSSRSETKLCYGYCKNVMRGCSAEYAGLLDSPWSGVVDSLNNLVTTHILSDTGIINVIKHLQTYFSEAIMAAMHNGPELEKKVKKTCGTPSLTPYSSGEPDARPPPHKNNVKWATDPDPGMVLFLSTIDKSKEFYTTIVDNFCDEQQHSRDDHSCWSGDRFGDYTQLLINPGTDSQRYNPEVPFNAKAQTGKLNELVDKLFKIRKSIGAAAPSNSIQTTHDIQNDMGEGAGGGEGQIGDDEEEYGGAHGAGDGAGDGPHTPIEESEGTTTNEVESRDAGKTAGSNPLEGTATWMLLTLVTMLFSSCS

Dlp[deltaGAG]

MLHQQQQQQHLHCRRKATATTTARLVIFSSPLLLLLLTTHLPPTLQADNGPAPQVAALAAPNPAGGVAGSSIIDQFSPNCSAVTHIFQARGIDAIEIPQKPSNERVLRYCESPSVGTCCTYNMETRMAMQSRQQLEGHTKDQISRMSGILGSKATKFKDIFTALLKESRTQFNSMFIRTYGVIYERNSYVFSDLFKELETYFANGRVDLLEVMDKFFNTLYQKMFTVLNTQYTFDENYMRCVSEHMKELKPFGDVPDKLSVQIKRSFVATRTYGQALTTASEVAKKVLNVRLNADCTGALTKMQHCGACKGYTEKPCTNYCVNVIKGCLHYQHEFDSEWENFAMAMDKVAERLLGSFNIVMVVEPLNIKISEAIMNFQDSGQDITNRVFQGCGRPKLKKMKRSISPKLQGVQILNARSPVEADTLDIDETLDEAIVLRERRAAEPGSQETSAQQSQEQGVGKSGNGGGGGGGNNRRQQQRRKQQQQRRKQQNNRDDNDDDDNESGGGREPILDRIVRDIRQRVKDYKKFWSNLPHSVCSNEDIASSSDVDGMCWNGHTIDRYMHSITTEHGSNPEFTGNPASTKQTAQMASQLSHLKNAIVHLRNAYNGQDVEWSEQEELPYAGAGAGAGAGSEDDEDDDEGAGLGPFEPSHKPDVERPSVDADNDDDEDAGGRGHMPTHTSRPTAGVDDKNPLIHTTHFDQDHNDLDEDHRQLDEDEDTDAGHDGANDNRSSDAPEKMTLRRALVVYLLPLYMAWFGGVCADLL

**Figure S1. Amino acid sequences of Dally^∆GAG^ and Dlp^∆GAG^.**

Amino acid sequences of Dally^∆GAG^ (top) and Dlp^∆GAG^ (bottom) are shown. Potential GAG attachment Serine residues in wild-type Dally (amino acid number: 549, 569, 573, 597, and 601) and Dlp (625, 629, 631, 643, and 686) are substituted to Alanine residues (red).


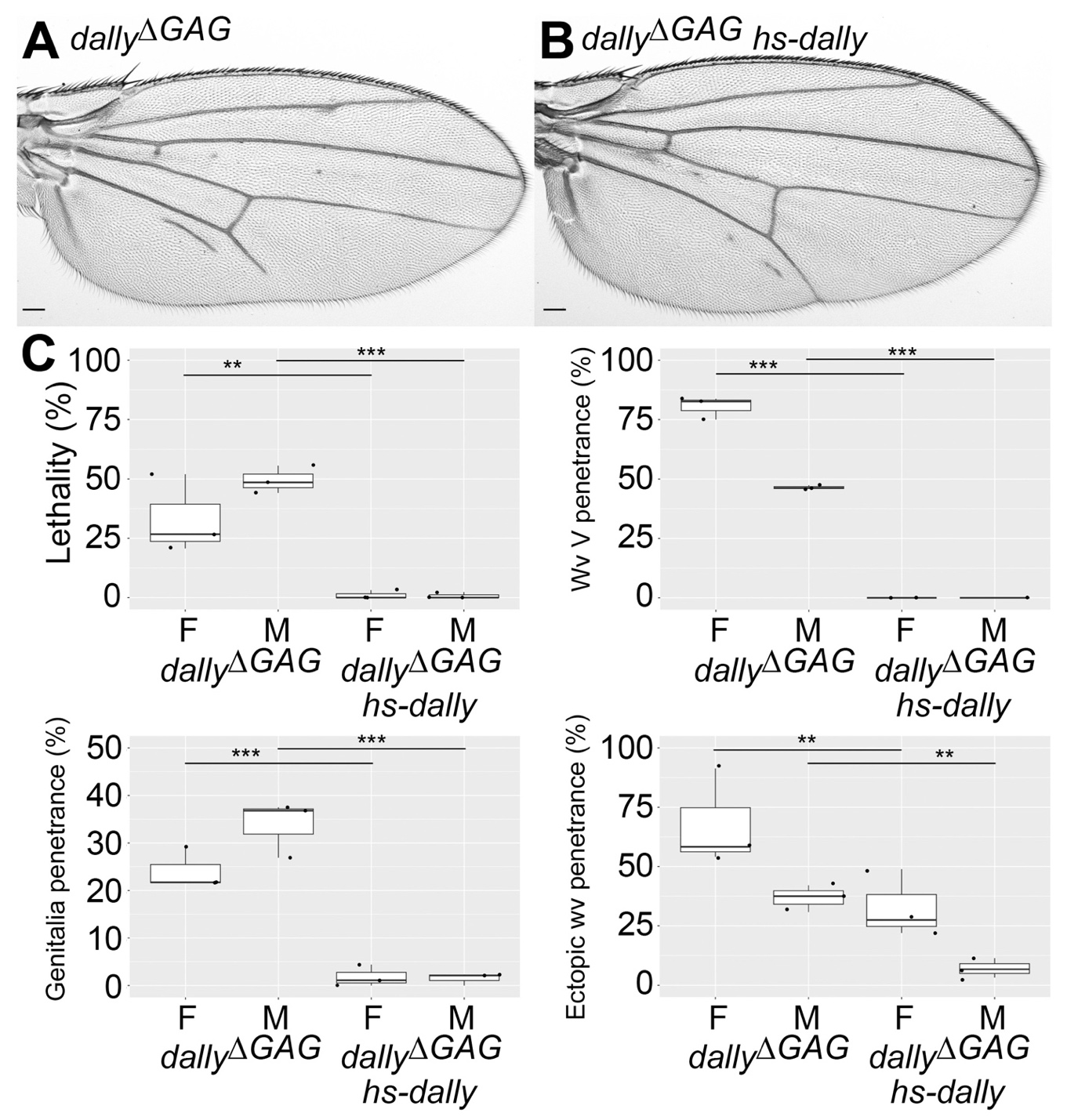


**Figure S2. Rescue of *dally^∆GAG^* mutant phenotypes by *hs-dally*.** (A and B) Wings are shown for *dally^∆GAG^*/*dally^∆GAG^* (A; *dally^∆GAG^*) and *dally^∆GAG^* *hs-dally/dally^∆GAG^* (B; *dally^∆GAG^ hs-dally*) adult females cultured at 29°C. (C) Quantification of lethality and adult phenotypes of *dally^∆GAG^*/*dally^∆GAG^* (*dally^∆GAG^*) and *hs-dally* *dally^∆GAG^*/*dally^∆GAG^* (*dally^∆GAG^ hs-dally*) adult females (F) and males (M) cultured at 29°C. Boxplots show summaries of three sets of independent experiments for the lethality (top left), penetrance of wing vein V defect (top right), genitalia defect (bottom left), and posterior ectopic wing vein phenotype (bottom right). n=343 for *dally^∆GAG^* females; n=346 for *dally^∆GAG^* males; n=572 for *dally^∆GAG^ hs-dally* females; n=576 for *dally^∆GAG^ hs-dally* males. Scale bars: 200 μm. ***P*<0.01; ****P*<0.001.


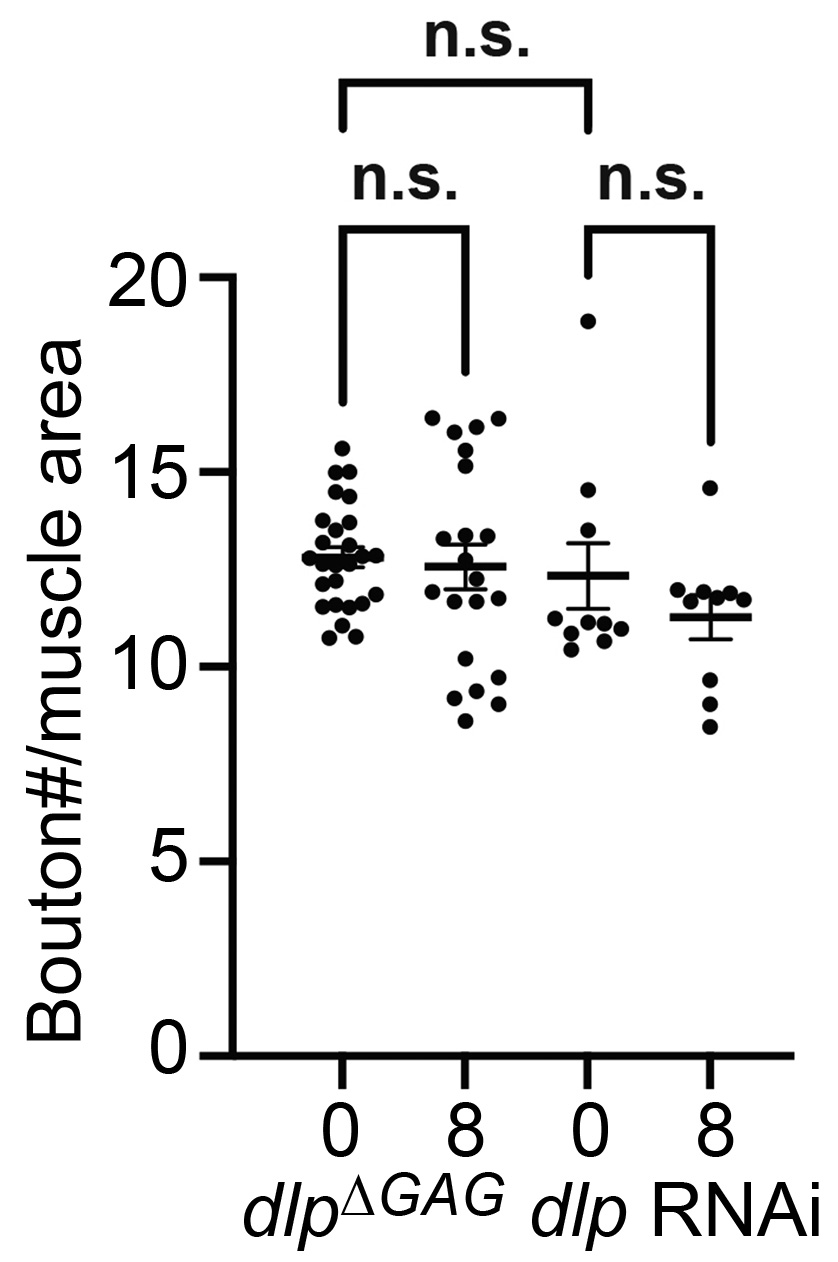


**Figure S3. Comparison of the NMJ phenotype between *dlp^∆GAG^* and *dlp* RNAi animals.** Quantification of bouton number per muscle area (10000 μm^2^) in the NMJ of *dlp^∆GAG^* and *dlp* RNAi animals (*UAS-dlp RNAi/+; 24B-Gal4/+*) at 0 or 8 hours of starvation. Data for RNAi animals was obtained from our previous publication (53) and data for *dlp^∆GAG^* is the same as Fig. 5E. n=26 for *dlp^∆GAG^* hour 0; n=21 for *dlp^∆GAG^* hour 8; n=10 for *dlp* RNAi hour 0; n=10 for *dlp* RNAi hour 8. n.s. not significant.
